# Biochar for Wastewater Treatment: Preparation, Modification, Characterization, and Its Applications

**DOI:** 10.3390/molecules30214288

**Published:** 2025-11-04

**Authors:** Ababo Workineh Tadesse, Mingjie Huang, Tao Zhou

**Affiliations:** 1 Hubei Key Laboratory of Multi-Media Pollution Cooperative Control in Yangtze Basin, School of Environmental Science and Engineering, Huazhong University of Science and Technology, Wuhan 430074, China; abiyework@gmail.com; 2 Department of Natural Resource Management, College of Agriculture and Veterinary Sciences, Ambo University, Ambo P.O. Box 19, Ethiopia

**Keywords:** pyrolysis, biochar, adsorption, hydrothermal carbonization, wastewater, heavy metal

## Abstract

Water contamination has become a critical issue, resulting in a decline in global water quality that harms both human health and the environment. Biochar, a porous carbon-rich material produced through the thermochemical decomposition of biomass, has attracted significant attention in wastewater treatment. This review provides a comprehensive overview of biochar preparation methods, modification strategies, characterization techniques, and environmental applications in wastewater treatment. Relevant information was gathered from peer-reviewed articles published in reputable databases. Among various production methods, pyrolysis is the most commonly employed technique for biochar production. The type of biomass and operational conditions, such as residence time, heating rate, and temperature, significantly impact the yield, structure, and composition of biochar. Advanced techniques, including FTIR, XRD, XPS, SEM, TGA, and BET, play a crucial role in characterizing biochar and evaluating its potential environmental applications. This review emphasizes the effectiveness of biochar in removing organic and inorganic contaminants from wastewater and highlights its growing role in environmental remediation. However, there is an ongoing debate regarding its long-term stability and potential release of adsorbed pollutants, which may pose risk if not properly managed. Therefore, this review suggests that future research should also consider its environmental safety, lifecycle sustainability, and regeneration potential. Moreover, establishing quality standards and a regulatory framework for safe and effective biochar use is important to ensure its role as a long-term solution for sustainable water management.

## 1. Introduction

The world has been suffering and changing drastically due to human beings’ reckless use of natural resources [1]. As industry and human activities expand, a lot of environmental pollutants are released into the ecosystem [2]. These pollutants include persistent organic pollutants (POPs) such as organochlorine pesticides (OCPs), polyaromatic hydrocarbons (PAHs) [3,4,5], polychlorinated biphenyls (PCBs) [6,7], volatile organic compounds (VOCs), antibiotic resistance genes (ARGs) [8,9,10], endocrine-disrupting compounds (EDCs), and heavy metals (such as Pb, As, Hg, Cu, Cr, Cd, Ni, Ag, etc.) [11]. In addition, nutrients such as nitrates, sulfides, ammonia, and phosphates also contribute to environmental pollution [12,13]. These contaminants have the capacity to travel long distances and enter the food chain, ultimately accumulating in human and other animals [2,14]. Due to their persistence and bioaccumulation properties, these pollutants pose significant risks to human health, animals, the ecosystem, and biodiversity [1,15].

Water is a vital resource for sustaining life on earth [1]. However, pollution of this resource has increased significantly, making water pollution a critical global issue [14]. Over the past few years, the situation has worsened, leading to a continuous decline in global water quality [16]. This water quality decline poses a threat to human health, aquatic ecosystem, and other biodiversity [17]. Human activities, such as agriculture, manufacturing, improper waste management, industrial discharge, and the use of various chemicals, are the primary causes of pollution [18]. According to the United Nations World Water Development report, about 80% of municipal and industrial wastewater is discharged into the environment without adequate treatment. Thus, effective wastewater monitoring and treatment strategies are essential to mitigate pollution and protect water resources [19].

Various techniques have been employed to remove pollutants from wastewater, including filtration, coagulation, flocculation, membrane technology, plasma-activated treatment, chemical precipitation, reverse osmosis, and electrochemical treatment [20] However, these conventional methods are often ineffective in removing low-concentration pollutants and are associated with high operational cost, and produce secondary pollution [17]. Consequently, researchers are exploring more sustainable and eco-friendly alternatives for wastewater treatment. Among these, the application of biochar has gained significant attention [21,22]. Biochar is a carbon-rich substance produced from biomass feed stocks such as agricultural residue, animal manure, waste paper, sludge, and other materials [14]. This porous carbonaceous substance results from the thermochemical decomposition of biomass feedstock in the presence of little or no oxygen [23]. Owing to its eco-friendly nature, ability to eliminate a wide range of pollutants, cost-effectiveness, and straightforward preparation method, biochar has emerged as a promising material for wastewater treatment and environmental remediation [24].

Biochar possesses unique physicochemical properties, including a high specific surface area, a porous structure [17], high adsorption and ion-exchange capacities, regeneration ability, and structural stability [17]. These characteristics have made it highly attractive for various environmental applications. These applications include soil improvement [25,26], composting [24], carbon sequestration [26,27], providing green energy to replace fossil fuels [24] or bioenergy production [28], acting as a catalyst and adsorbent, lower greenhouse gas emissions [27] and wastewater treatment; biochar also has strong potential for removing diverse pollutants such as heavy metals [29,30], dyes [31], antibiotics like tetracycline [32], endocrine-disturbing compounds [33], and other organic and inorganic pollutants [34]. Owing to its broad applicability and environmental benefit, biochar is regarded as a promising and sustainable material for addressing environmental challenges [14,35].

**Figure 1:**
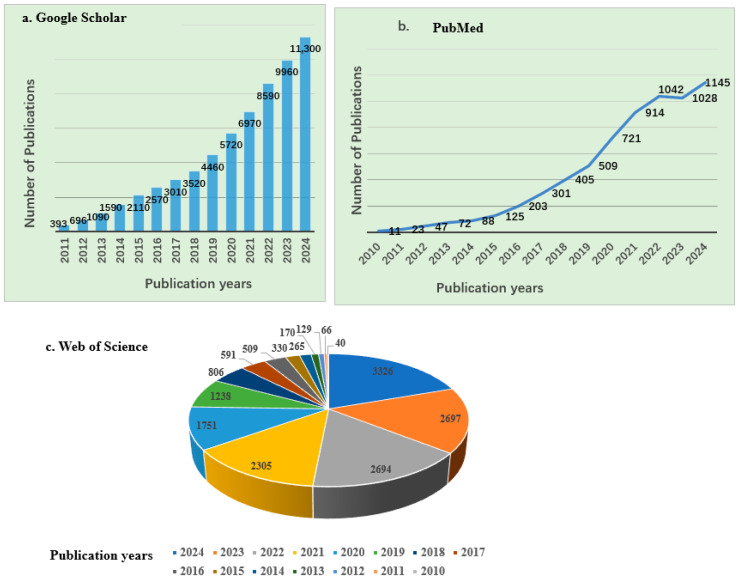
Number of publications on biochar and its applications between 2010 and 2024 (source: (**a**) Google Scholar (**b**) PubMed (**c**) Web of Science).

**Figure 2:**
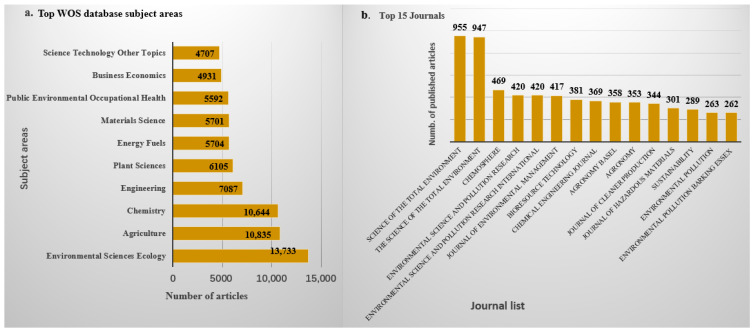
(**a**) Top subject area and (**b**) top journals that publish on application of biochar from WOS database.

Research on the application of biochar across various fields, such as wastewater treatment, soil remediation, and renewable energy production, is ongoing. According to Google Scholar and PubMed, approximately 61,979 and 6634 articles, respectively, have been published over the past decade and a half, highlighting a significant increase in research interest since 2010, as shown in Figure 1. Similarly, data from the Web of Science database indicated that from 2010 to 2024, about 16,918 articles related to biochar and its applications were published, covering a wide range of disciplines, mainly including Environmental Sciences, Ecology, Agriculture, Chemistry, Engineering, Plant Sciences, Energy Fuels, Materials Science, Public Environmental Occupational Health, Business Economics, etc. As shown in Figure 1, there has been a continuous upward trend in biochar-related publications. The top 15 journals contributing to these research fields are presented in Figure 2b, with Science of the Total Environment ranking first. The analysis showed that biochar use for environmental remediation is a widely recognized research area, with contributions from many countries. Based on publications from different countries retrieved from the Web of Science database from 2010 to 2024, China has contributed the most publications on biochar, accounting for 67.77% of the total, followed by the United States (Table 1).

Biochar can be produced through various thermochemical conversions, methods including pyrolysis, hydrothermal carbonization, gasification, microwave heating, and flash carbonization [2,30]. The yield and quality of biochar are influenced by various parameters such as types of biomass, reaction temperature, heating rate, residence time, particle size, and reaction environment [14]. Likewise, the pollutant removal efficiency of biochar depends on multiple factors, including pH, presence of coexisting contaminants, initial pollutant concentration, types of functional groups, pore structure, surface cation exchange capacity, biochar dosage, and production process [36]. Despite extensive research on biochar preparation and its applications, a significant knowledge gap remains regarding the impacts of feedstock types on biochar physicochemical properties, yield, quality, and pollutant removal efficiency in wastewater treatment. Therefore, the primary objective of this review is to summarize the current literature on biochar production, activation methods, examine the factors affecting its structure and functional characteristics, and evaluate its applications in wastewater treatment. Additionally, this review also outlines the factors that influence biochar removal efficiency in wastewater treatment.

## 2. Materials and Methods

**Figure 3:**
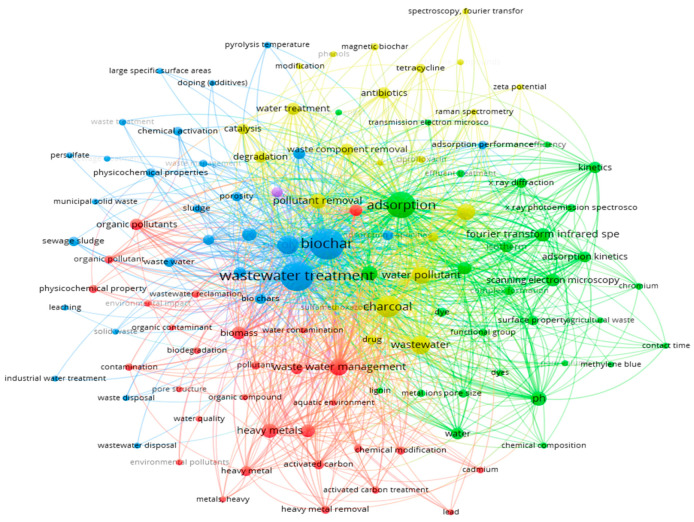
Keywords used to search biochar-based articles for this review.

A comprehensive literature search was conducted, reviewing 113 peer-reviewed articles published between 2010 and 2024, sourced from reputable databases such as Web of Science, PubMed, Science Direct, and Google Scholar. The literature search used keywords including biochar preparation, biochar activation, adsorption, wastewater treatment, pyrolysis, hydrothermal carbonization, organic and inorganic removal, and others. To identify research trend and relationships, VOS viewer software was used to perform keywords co-occurrence analysis of the collected studies. Figure 3 highlights the most frequently used keywords related to biochar applications in wastewater treatment.

### 2.1. Biochar Preparation Methods

#### 2.1.1. Pyrolysis

**Figure 4:**
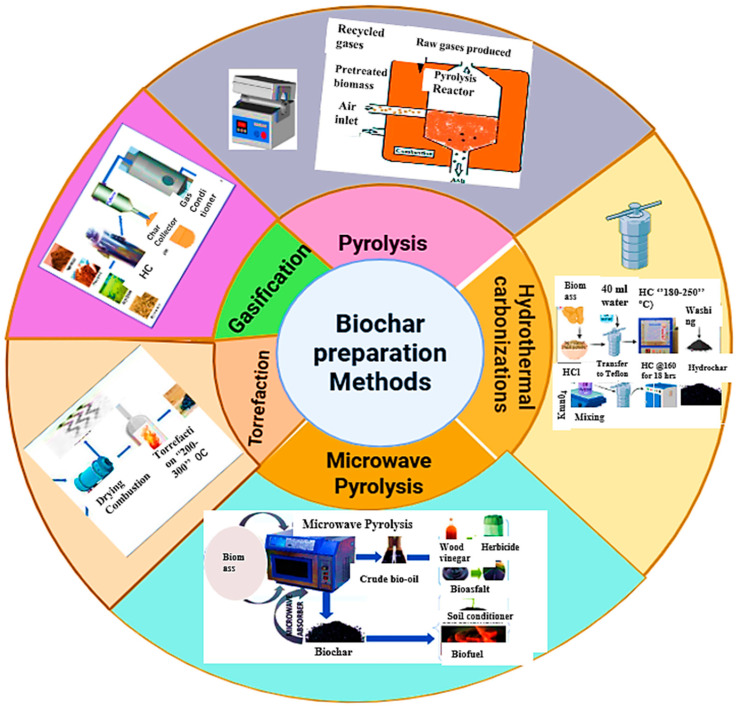
Biochar preparation methods.

Biochar is a stable carbon-rich material made from sources such as animal manure, agricultural and forestry wastes, sewage sludge, bioenergy crops, and other organic residues. It is produced by thermochemically decomposing these materials in a controlled or low-oxygen environment [37,38]. Among the production methods, pyrolysis is the most widely employed. In this process organic compounds are thermally broken down in an oxygen-free environment at a temperature ranging from 250 to 900 °C, converting biomass into higher-value products, such as biochar, syngas, and bio-oil [24]. The type of biomass influences biochar yield, while temperature primarily determines the efficiency of the process [24,39]. As reported by Yaashika et al. [24], increasing pyrolysis temperature reduces biochar yield, but increases syngas production. Depending on the heating rate, temperature, pressure, and residence time, pyrolysis can be categorized into slow, intermediate, and fast pyrolysis [40]. Figure 4 shows the main biochar preparation techniques, including pyrolysis, hydrothermal carbonization, torrefaction, gasification, and microwave pyrolysis.

Fast pyrolysis is a thermochemical process that operates at temperatures between 500 and 1000 °C with the potential to convert solid biomass into liquid bio-oil [25]. Due to rapid heat transfer and fine particle formation, fast pyrolysis typically yields more bio-oil than biochar [41]. The process is characterized by a heating rate that exceeds 100 °C per minute, and very short residence time, usually between 0.5 to 2 s. According to Yaashika et al. [24], a key feature of fast pyrolysis is maintaining the fume residence time in the hot zone to achieve high-quality bio-oil. At high temperatures, particularly around 1000 °C, a larger proportion of the final product shifts towards biogas formations. When the temperature exceeds this range, flash pyrolysis occurs, characterized by an extremely high heating rate of 100 °C per minute and a very short residence time, usually less than 0.5 s [41]. The pyrolysis temperature significantly influences the physicochemical properties of biochar. Biochar with large surface area, hydrophobicity, microporosity, low polarity, acidity and aromaticity is produced by high pyrolysis temperatures. This type of biochar is more effective at removing nonpolar organic contaminants, due to hydrophobic interactions facilitated by O-bearing functional groups. Conversely, slow pyrolysis, which operates at lower temperatures and longer residence time, produces greater biochar yields with smaller surface areas and higher oxygen content. These characteristics enhance its ability to remove inorganic contaminants through ionic interaction. Enaime et al. [20] further reported that high-temperature pyrolysis typically yields more hydrophobic biochars with larger surface areas and greater micropore volume, making it particularly more effective for the sorption of organic pollutants. Conversely, low-temperature biochar has a lower surface area, smaller pores, and more oxygen-containing functional groups, which enhance its effectiveness in removing inorganic contaminants [34,42].

Moderate pyrolysis is a thermal decomposition process carried out at an intermediate temperature (300–500 °C) in the absence of oxygen. This temperature range lies between that of fast pyrolysis and slow pyrolysis. The process produces a balanced mixture of biochar, bio-oil, and gas (see Table 2). This process prevents the formation of higher molecular weight tars and promotes the synthesis of fine biochar, bio-oil, and syngas. According to Ravindiran et al. [41], the shape and size of biomass have less significant impact in moderate pyrolysis compared to other thermochemical methods. It has been reported that pyrolysis is the most common technique for preparing biochar, and has recently emerged as a cutting-edge alternative to liquefaction, gasification, and incineration technologies for effective resource recovery [43]. As summarized in Table 2, different thermochemical conversion techniques exhibit distinct temperature ranges, reaction times and product distributions. Slow pyrolysis and torrefaction, which operate at lower temperatures, result in a higher formation of solid biochar, whereas fast pyrolysis and gasification yield less solid biochar and greater bio-oil and syngas. Hydrothermal carbonization performed under wet conditions achieves a higher solid yield and produces hydrochar. These comparisons highlight trade-offs among residence time, temperature, and product yield, offering insight into selecting appropriate conversion techniques for specific environmental or energy-related applications.

#### 2.1.2. Hydrothermal Carbonizations

It is the biochar preparation process involves heating biomass 200–250 °C under high pressure while suspended in liquid for a few hours. This method is suitable for wet feed stocks and can produce liquid, solid, and gaseous products [37]. The term ’wet pyrolysis’ refers to hydrothermal carbonization. The main advantage of hydrothermal carbonization is that it does not require intensive energy input before or during the processing, making it more energy efficient than other methods [40]. The process yields hydrochar, which differs from the product of dry pyrolysis. Unlike pyrolysis, hydrothermal carbonization produces more biochar and eliminates the need for a drying stage. Water plays a crucial role in this process, hydrolyzing the glycosidic bonds in biomass, particularly those in cellulose and hemicellulose, to create more porous hydrochar. However, despite its advantage water is not ideal for feedstocks with rapid reaction rates or prone to drastic changes, which can complicate process control [44].

#### 2.1.3. Gasification

Gasification is a powerful thermochemical technology that changes carbon-rich material into syngas, under high temperatures (800–1000 °C) and with limited oxygen. Compared to other biochar production methods, gasification yields less biochar and more syngas [37]. Gasification involves applying an oxidizing atmosphere by adding carbon dioxide, air, oxygen or mixtures as gasification agents, leading to partial combustion and formation of syngas. Syngas produced mainly consists of CO, CO_2_, H_2_, and CH_4_, which can be used for electricity generation, chemical production, and the production of synthetic fuel. During gasification, a small amount of biochar (10%) is produced while a significant portion (85%) is converted into gas [40]. Additional byproducts, such as hydrogen, carbon dioxide, methane, nitrogen, and acetylene, can be produced depending on the gasification agent. Hydrogen production peaks when steam is used as the gasification agent with a high heating value [41]. According to J. Makwana et al. [45], about 56–59% (vol.) of the produced gas is nitrogen when air is used as the gasification agent. They also noted that using oxygen and steam as gasification agents yields 30–34% (vol.) and 24–50% (vol.) hydrogen gas, respectively (Table 3). Temperature control is a challenge of the gasification process, as it requires significant amounts of air and oxygen. Compared to other conversion processes, gasification produces substantially less biochar relative to syngas, which leads to higher greenhouse gas emissions [41]. The overall gasification process can be affected by the type of gasification agent, biomass composition, pressure, and reaction temperature [26]. Among these, temperature is the most critical factor [46]. Their findings showed that increasing temperature increases the production of CO, H_2_, and C, while decreasing CO_2_, CH_2_, hydrocarbons, and tar formation. Table 3 compares the gas composition produced with various gasification agents. The type of gasification medium significantly affects the relative proportion of the produced gas, such as hydrogen, carbon dioxide, and methane syngas. From the following table, it is possible to conclude that the gasification agent has a significant impact on the quality and contents of syngas produced. The air gasification agent produced the lowest hydrogen content, which might have resulted from nitrogen dilution, while the steam and CO_2_ gasification agents produced higher hydrogen content. Thus, hydrogen production and calorific value of the produced syngas can be greatly increased by selecting the right gasification medium.

#### 2.1.4. Flash Carbonization

Flash carbonization is a rapid process that produces biochar in less than 30 min. It operates at high temperatures (300–600 °C) and under elevated pressure (1–2 MPa) at the bottom of a packed bed biomass [39]. Unlike pyrolysis, which occurs in an oxygen-free environment, flash carbonization uses a controlled amount of oxygen or air to partially burn of some of the biomass. During the process, air flows down through the bed, while the combustion front rises upward, ensuring uniform carbonization. The process supplies about 0.8–1.5 kg of air is supplied per kilogram of biomass. This method produces solid biochar, and gases as byproducts [39].

#### 2.1.5. Torrefaction

Torrefaction is a thermochemical treatment method that produces a hydrophobic solid product with a low O/C ratio by removing carbon dioxide, oxygen, and moisture from biomass under inert conditions at 200–300 °C with a heating rate of 50 °C per minute. This process transforms biomass into biochar, bio-oil, and syngas with a moderate residence time of 20–40 min [41]. During torrefaction, the long polysaccharide chains in the biomass are also depolymerized. Because this method is carried out at a slow heating rate, this technique is sometimes referred to as a slow heating rate [26]. The biomass moisture content is eliminated during this process, and cellulose, hemicellulose, and lignin undergo partial degradation. Furthermore, instead of producing liquid or gaseous byproducts, torrefaction yields biochar as a solid product.

According to Ravindiran et al. [41], torrefaction can produce high-quality biochar with a low oxygen-to-carbon ratio, high energy density, and hydrophobicity. They noted that a low oxygen-to-carbon (O/C) ratio, typically ranging from 0.2 to 0.6, preferably around 0.4, is an indicator of high-quality biochar. The O/C ratio determines the carbon stability of biochar since a greater ratio causes more oxidation and limits carbon lost as carbon dioxide, while lower a O/C ratio increases the stability of biochar, and if the ratio is less than 2, the biochar half-life can reach 1000 years. Nevertheless, the torrefaction process provide biochar with an O/C ratio higher than 0.4, which can lead to a low-quality biochar [41].

Lin et al. [17] reported that, due to its high carbon content and calorific value, as well as its low production costs and energy consumption, torrefied biochar has the potential to serve as an alternative renewable fuel. However, the performance and quality of torrefied biochar was inconsistent, and further modification and optimization of the processes. To address this, it is important to consider different types of torrefaction available, including dry torrefaction, wet torrefaction, steam torrefaction, microwave torrefaction, and oxidative torrefaction [17]. For instance, according to their report, the reaction atmosphere in dry torrefaction has shifted from an inert gas to a non-inert gas atmosphere, such as O_2_, CO_2_, and NH_3_, with nitrogen serving as a conventional carrier gas. In contrast, wet torrefaction technology is suitable for wet or moist biomass such as sewage sludge, algae, and animal manure due, to its ability to minimize the energy-intensive process required for the pre-drying of high-moisture feedstock [47]. Moving to another technique, steam torrefaction involves pretreating biomass with high-temperature and high-pressure steam explosions, where heat transfer is affected by steam. Furthermore, microwave torrefaction utilizes a microwave reactor to direct electromagnetic radiation, allowing for a deeper penetration depth and a more consistent temperature distribution, which is favorable for the consistent formation of biochar [17]. The comparison of different torrefaction methods (i.e., dry, wet, steam, and microwave), their operating conditions, and solid yields is summarized in Table 4. These methods differ in terms of time, temperature, pressure, and moisture content, which determine the product quantity and quality. Dry torrefaction is carried out at a temperature of 200–300 °C, resulting in a higher solid yield than the others. However, biomass must be pre-dried prior to the process. Wet and steam torrefaction takes place at elevated pressure, enabling the treatment of moist biomass directly without pre-drying, but they produce lower solid yield. Microwave torrefaction occurs in a short time (2.5–5 min) and produces a moderate yield (Table 4).

## 3. Biochar Characterization and Its Properties

### 3.1. Biochar Characterizations

Biochar characterization is crucial for understanding its properties, optimizing production, and tailoring its applications. Additionally, biochar characterization is essential to determine its suitability and performance for various environmental applications [43]. The key parameters of biochar characterization include its structural morphology, surface functional groups, porosimetry analysis, thermal stability, ash content, and elemental composition [24]. Pyrolysis conditions, such as temperature variation, contact time, and the introduction of chemicals, can further enhance and tailor the physical and chemical properties of biochar [48]. Currently, a variety of advanced methods are employed to characterize biochar, including scanning electron microscopy (SEM), X-ray diffraction (XRD), thermogravimetric analysis (TGA), Fourier transform infrared spectroscopy (FTIR), nuclear magnetic resonance spectroscopy (NMR), Brunauer–Emmett–Teller analysis (BET), Raman spectroscopy, and Field Emission Gun Scanning Electron Microscope (FEG-SEM) [24,43].

**Figure 5:**
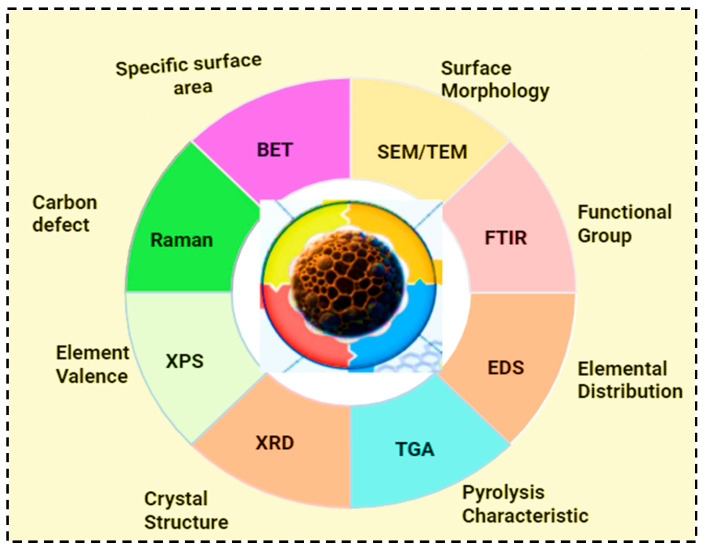
Characterizations of biochar.

The surface structure of biochar is commonly analyzed using scanning electron microscopy (SEM), which provides detailed images of pore organization, including distribution of microporous and mesoporous. SEM is also used to examine surface morphological change during the adsorption process [24,33,43]. X-ray diffraction (XRD) is a widely accepted method for determining biochar structure and crystallinity. Thermogravimetric analysis (TGA) assesses the chemical and physical properties of biochar as temperature increases. Fourier transform infrared spectroscopy (FTIR) identifies the surfaces functional groups present on biochar [1]. The structural composition and degree of carbonization of biochar can be analyzed using nuclear magnetic resonance spectroscopy (NMR). These techniques are further employed to evaluate the stability and chemical transformation of biochars. The Brunauer–Emmett–Teller analysis (BET) method measures the surface area of biochar [24,33]. Raman spectrometry is a highly effective form of subatomic spectrometry that identifies the molecular and structural properties of biochar. X-ray photoelectron spectrometry (XPS) assists in determining the elemental composition, surface chemistry, and bonding configuration of biochar [24]. It is also used for identifying and quantifying functional groups of biochar [33]. Finally, the Field Emission Gun Electron Microscope is employed to evaluate structural alterations in biomass-driven biochar samples with high resolution [43]. The key techniques used for biochar physicochemical characterizations and the corresponding properties they reveal are shown in Figure 5. These methods highlight the complementary insights that are important for understanding the structure, composition, and functionality of biochar.

### 3.2. Biochar Properties

#### 3.2.1. Physical Properties

Biochar is a carbon-rich material produced through pyrolysis, a process that involves heating organic material in an oxygen-limited environment [23]. Owing to its potential to improve soil health, sequester carbon, and support sustainable farming practices, it has garnered significant interest [23]. The physical properties of biochar can be influenced by the type of biomass, and thermochemical conditions (such as pyrolysis such as temperature, heating rate, residence time, and biomass pretreatment method) [35]. Particle size, pore size, surface area, density and pore volume are the key physical properties of biochar [41]. Among these pyrolysis temperature plays a critical role as increasing temperature leads to the expulsion of moisture and volatile compounds, resulting the formation of pores on the biochar surface [49]. The original structure of biomass, such as fracture development, microstructural rearrangement, and attrition, can be altered during pyrolysis processes [35]. Biochar is characterized by its high surface area and porosity, low bulk density, variable and irregular shapes [23]. Porosity and surface area are among the most crucial physical properties that determine the amount of active compounds in biochar, which are used to enhance biochar properties such as cation exchange capacity, water holding capacity, and adsorption performance [25]. Furthermore, biochar has a complex pore structure, including macropores, mesopores, and micropores, which can provide a large surface area and high adsorption capacity [17].

#### 3.2.2. Chemical Properties

Understanding the chemical characteristics of biochar helps to tailor its properties for specific applications. The type of biomass and pyrolysis conditions during production determine the chemical characteristics of biochar [18]. The key chemical properties of biochar include carbon and ash content, pH, cation exchange capacity, elemental composition, and functional groups. These characteristics helps to know why biochar aids in removing environmental pollutants [50]. The surface of biochar contains various oxygen-containing functional groups, such as carboxyl, hydroxyl, phenolic hydroxyl, and carbonyl groups [37], which can affect nutrient retention, pH, and interactions with soil organic matter interaction [14]. These characteristics are the key determining factors that affect the movement, bioavailability, and transformation of pollutants [37].

**Figure 6:**
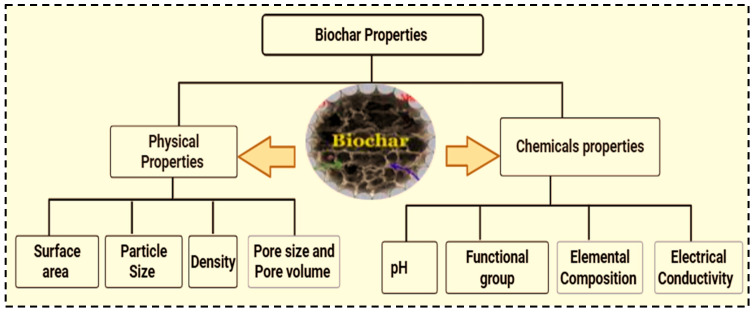
Biochar physical and chemical properties.

The cation exchange capacity (CEC) of biochar is notably high, particularly after aging or weathering conditioning. This property allows biochar to retain positively charged nutrients such as potassium, magnesium and calcium As a result, it reduces nutrient leaching and increases nutrient availability to plants over time [50]. The carbon content of biochar ranges from 50 to 90%, indicating its degree of carbon stability. This stability makes it resistant to microbial decomposition, thereby serving as long-term carbon storage option. The pH of biochar which varies depending on pyrolysis temperature and feedstock strongly influences its adsorption capacity and the presence of active adsorbent sites on its surface [50]. Additionally, the presence and proportion of oxygen, carbon, hydrogen, and nitrogen significantly affect the chemical properties of biochar. Numerous functional groups are formed during pyrolysis, further improving biochar’s chemical properties. Figure 6 summarizes the key biochar properties that influence its performance and functionality.

## 4. Factors Affecting Biochar Properties

The properties of biochar are mainly affected by various factors, including feedstocks, temperature, particle size, heating rate, moisture conditions, residence time, treatment process, and reactor types [51]. These factors not only impact the quality of biochar but also the yield [24]. A comprehensive understanding of these factors is essential for optimizing biochar production and tailoring its physicochemical properties for specific applications. The following sections discuss each of these factors in detail.

### 4.1. Feedstocks

The type of feedstock’s greatly influence both biochar yield and quality [21,52]. Biochar produced from different feedstocks exhibits different physicochemical characteristics due to variation in their elemental composition and biochemical structure. For example, compared to wood derived biochar (349 mg/kg), straw-derived biochar had a greater potassium content (961 mg/kg) [21]. Kolodynska et al. [53] reported that biochar produced from cow and pig manure shows different elemental contents. These findings indicate that the physicochemical properties of biochar are significantly influenced by the types of feedstock used [21,54]. Feedstock with higher volatile matter content such as straw tend to yield a lower amount of biochar, as volatile components are more easily released during pyrolysis [21]. The ash content, composition, and structure of feedstocks vary, which affects the pyrolysis process, as well as the physicochemical and functional properties of biochar [55]. A wide range of feedstocks sources are available for biochar production, including woody biomass, aquatic biomass, paper waste, urban garbage, and agricultural biomass [56]. Common agricultural feedstocks include rice straw, cotton stalk, coconut shells, etc., while municipal wastes such as paper mill and sewage sludge are the most common. Pine sawdust and wood chips are the most typical woody biomass, whereas aquatic seaweed and macroalgae species are common aquatic waste, frequently used for biochar production [56].

Depending on the source materials, the most common biochemical components of raw feedstocks are hemicellulose at 15–60% (C_5_H_8_O_4_), cellulose at 20–60% (C_5_H_8_O_4_)_m_, and lignin at 5–40% [C_9_H_10_O_3_(OCH_3_) 0.9–1.7]_n_ [55]. Variations in cellulose, lignin, hemicellulose, and mineral content also cause different feedstock types to react differently to certain pyrolysis conditions [55]. Consequently, pyrolyzing diverse feedstocks at the same temperature can produce biochars with distinct physicochemical properties. Therefore, choosing the right feedstock ingredient and optimizing pyrolysis conditions are critical steps in designing biochar tailored for specific applications [57].

During the pyrolysis process, cellulose and hemicellulose primarily contribute to the formation of aromatic rings and oxygen-containing functional groups in biochar. The two components breakdown more quickly than lignin whose breakdown is more complex and occurs over a wider temperature range [58]. Feedstocks, with high cellulose and hemicellulose tend to generate different oxygen-containing functional groups; however, biochar yields remain limited. Conversely, feedstock with higher lignin content results in higher biochar yield due to the thermal stability of aromatic monomers and high fixed carbon. The high fixed carbon and thermal stability of aromatic monomers, as opposed to the aliphatic carbon phases of cellulose and hemicellulose, result in a higher output of biochar when lignin is present in greater proportions.

Biomass is a complex solid material derived from biological, organic, or inorganic material and can be classified as: woody and non-woody biomass [24]. Woody biomass includes forestry and tree residue, while non-woody biomass contains agricultural residue, animal waste and industrial waste etc. Among various feedstock characteristics, moisture content has a significant influence on biomass formation. It can be present as liquid water, water vapor, or moisture absorbed in the biomass pores. High moisture content in biomass mainly prevents char formation and increases the energy required to reach the pyrolysis temperature. Because of the remarkable reduction in heat energy and the time needed for the pyrolysis process, low-moisture-content biomass is more suitable and economically viable for the production of biochar than high-moisture-content biomass [24]. According to Hasssan M. et al. [55], compared to biochar derived from manure (MBC) and grass (GBC), biochar made from hardwood (HBC) and softwood (SBC) exhibit higher surface area and carbon content, but lower oxygen and mineral content. The stability and aromaticity of biochar followed the order HBC > SBC > GBS > MBC. In addition to influencing biochar properties, the type of feedstock affects its adsorption capacity. Biochar produced from different precursors, such as agricultural residues, animal manures, forestry waste, and algal biomass, exhibits distinct physicochemical characteristics, including variations in surface functional groups, pore structure, surface area, and elemental content. These variations significantly affect biochar’s adsorption capacity. For instance, Hassan M. et al. [55] reported that grass and animal manure-derived biochar is more suitable for the removal of ionic contaminants compared to hardwood and softwood-derived biochar. They also mentioned that corn straw-derived biochar shows the greatest adsorption capacity than peanut shell and wheat straw-derived biochar for the removal of NH_4_^+^.

### 4.2. Temperature

The temperature at which pyrolysis occurs affects the physicochemical characteristics [59], and the structure of biochar, including its elemental composition, pore structure, surface area, and functional groups [1,60]. Higher temperatures promote the release of volatiles, which change these characteristics and also influence the yield and distribution of final products [24,55,61]. For instance, fast pyrolysis produces 60–75% bio-oil, 15–20% charcoal, and 10–20% gas products, indicating that higher temperatures favor bio-oil formation over biochar. In contrast, slow pyrolysis at 400–500 °C primarily produces biochar with only traces amount of gas and liquid [1]. The highest yields of bio-oil are obtained at temperatures between 400 and 550 °C, while bio-oil dispersion diminishes at temperatures above 600 °C; further breakdown processing turns the biochar and bio-oil into gas products. Temperature also affects the adsorption performance of biochar. For instance, Meng et al. [62], indicated that biochar prepared from pig manure for the removal of Cu at a temperature of 400 °C resulted in a specific surface area of 10.59 m^2^/g and adsorption capacity of 12.72 mg/g, respectively. Increasing the temperature to 700 °C results in a specific surface area of 73.49 m^2^/g and an adsorption capacity of 9.09 mg/g. Huang et al. [63] reported that rice husk-derived biochar prepared at 300, 500, and 700 °C with equal residence time for Cd removal from aqueous solution exhibits specific surface area of 21.37, 44.92, and 242.53 m^2^/g, with corresponding adsorption capacities of 62.75, 77.37, and 93.50 mg/g.

### 4.3. Residence Time

Residence time is the duration that biomass stays inside the reactor during the pyrolysis process [18]. It is one of the most critical operational factors affecting product yield and distribution [24,51]. The duration for which biomass is maintained at pyrolysis temperature plays a crucial role in determining the characteristics of the produced biochar. Slow pyrolysis usually takes place at temperatures between 300 and 600 °C with long residence times of 30 to 120 min, which promote higher biochar yields and more complete carbonization. Intermediate pyrolysis generally involves residence times of 5 to 30 min at 400–600 °C, resulting in a balanced mix of solid, liquid, and gaseous products. In contrast, fast pyrolysis occurs at 500–1000 °C with very short residence times of less than 2 s, maximizing bio-oil production while reducing char formation [18].

Organic matter is broken into gases and liquids by prolonged heating, leaving behind a more condensed carbon-rich residue [51]. As the residence time increases, the organic vapors produced from lignocellulosic biomass undergo secondary reaction that enhances char and increase carbon content. Conversely, shorter residence time results in biochar with less stability and a lower carbon content. Thus, an optimal residence time is essential to balance biochar yield, stability, and surface reactivity depending on the intended application [51]. The carbon structure of biochar becomes more aromatic due to prolonged heat exposure, increasing its resistance to microbial deterioration. Porosity and surface area of biochar typically increase with longer residence times. This enhancement occurs due to structural reorganizations and the gradual release of volatiles at high temperatures, which make the matrix more porous. Lou et al. [3] examined the impact of residence time on the pore characteristics and specific surface area of biochar and found that these properties increase up to 2 h at temperatures between 500 °C and 900 °C, but begin to decline thereafter. Notably, when the residence time at high temperature exceeded 2 h, both the specific surface area and the pore structure showed a significant reduction. A similar result was reported by [49], which indicated that extending residence time from 90 to 150 min lead to a decrease in surface area and pore volume of biochar. This finding suggests that the pore structure of the biochar collapsed further during the co-pyrolysis process as the residence time increased, resulting in a reduction in the biochar’s surface area. Longer residence durations also lead to higher ash content as organic materials break down, leaving behind inorganic minerals. At temperatures below 800 °C and 750 °C, longer residence times result in the production of more char and gas, respectively. Ningbo et al. [51] also noted that high temperatures combined with extended residence promote the formation of phenols in bio-oil. Six minutes of residence time at 900 °C can achieve 6.5% energy profit.

### 4.4. Particle Size

The choice of biomass particle size is crucial for effective pyrolysis and product yields [1]. The particle size of biomass feedstock significantly influences heat and mass transfer during pyrolysis, thereby affecting the yield and quality of biochar. Smaller particle size, typically below 1 mm, increases the surface area-to-volume ratio, promoting uniform heat transfer and accelerating pyrolysis by enabling higher heating rates. Rapid heating favors the generation of gas and liquid products over solid waste, which can lead to a lower yield of biochar [28]. In contrast, larger feedstock particles, generally above 5 mm, hinder efficient heat transfer, resulting in uneven carbonization and reduced overall biochar yield. Smaller particles enhance heat penetration and provide more uniform thermal exposure, leading to increased carbonization. In contrast, larger particles tend to produce biochar with lower fixed carbon content and higher volatile matter due to incomplete pyrolysis. Additionally, smaller particles yield biochar with greater porosity and surface area, making it more suitable for applications such as adsorbents, water filtration, and soil amendment. On the other hand, larger particles are less effective for these applications due to their lower porosity and surface area. It is important to note that smaller particles may also exhibit a larger ash content due to increased release of volatile chemicals and exposure to greater temperatures [1].

### 4.5. Pretreatment of Biomass

Pretreatment of biomass significantly influences the yield and quality of biochar. This is because the feedstock’s chemical, physical, and structural features are altered by these pretreatment procedures, which can affect both pyrolysis behavior and the final biochar properties [22]. Common pretreatment methods include reducing the particle size of biomass and immersing it in chemicals. Specifically, a large amount of biochar is produced when the size of biomass particles is reduced, due to an increase in surface area, which enhances heat transfer [56]. Furthermore, biomass harvesting time also affects its composition and the quality of the biochar produced. Early biomass harvesting can result in higher moisture levels and lower lignin content, which may reduce the energy content. In contrast, late harvesting increases lignin content but may decrease overall yield due to natural degradation [56].

## 5. Activation of Biochar

Biochar activation is the process of enhancing the properties of biochar to improve its performance for a specific purpose [14]. There are several drawbacks of using pristine biochar in terms of recycling and recovery. Its poor porosity and limited adsorption sites, make it less effective compared to modified or activated biochar. Therefore, to achieve higher adsorption efficiencies, biochar should be activated or modified. Thus, biochar activation is a crucial process to enhance its adsorption performance and physicochemical properties. Activation of biochar enhances its adsorption capacity by altering surface chemistry, functional groups, and pore structure. Biochar activation enhances biochar effectiveness in applications such as adsorption, pollutant removal, catalysis, nutrient retention, carbon sequestration, soil improvement, and greenhouse gas emission reduction [14,25]. Numerous techniques have been used to increase the functional groups of biochar, thereby boosting its adsorption capacity. Biochar can be activated through physical and chemical methods [41]. But not all activation methods are equally effective in improving the biochar adsorption capacity. Each method is discussed in the following Section 5.1 and Section 5.2.

### 5.1. Physical Activation

Physical activation involves using oxidizing agents such as CO_2_, steam, and air at temperatures between 350–1000 °C, which is also called gas activation [25]. According to Leng et al. [25], physical activation is carried out after pyrolysis. The most common physical activation methods include using steam, gas, ball milling, magnetic properties, and microwave-assisted activation. The steam generated during heating increases the biochar’s surface area and pore size, where the prepared biochar is heated between 700 and 900 °C [41].

For physical activation to be successful, several factors, including temperature, activating substances, and the degree of activation, play key roles. Physical activation uses very little energy and is reasonably priced. Biochar’s porous structure increases with rising temperature and air exposure. Air oxidation is widely recognized for its numerous benefits, such as easy access to air and the absence of chemicals, resulting in relatively little wastewater. However, the primary disadvantage of air is that, if improperly managed, it can cause pyrolysis to transition to combustion, releasing more heat and producing less biochar. Biochar heated to 800 °C in the presence of hydrogen gas is referred to as “heat-treated” biochar because heat is used as an activator to enhance its crystalline character and create carbon–hydrogen (C-H) bonds. The trapped tar in the pores can react with active oxygen agents, causing the pores to open. Furthermore, pores may grow and widen as a result of their subsequent reaction with the carbon skeleton, which produces CO and CO_2_ [25]. Physical activation, which typically uses either oxidizing gas such as CO_2_, steam, or air at 700–900 °C, is an important process for modifying the structural and surface properties of biochar [41]. Through physical activation, partial gasification of the carbon matrix increases the width of existing pores and creates micro- and mesopores, thereby increasing total pore volume and specific surface area. The newly created pore network encourages mass transfer and provides more available adsorption sites for contaminants. During physical activation, there is some limited surface oxidation occurs, which introduces oxygen-based functional groups such as -COOH, -OH, and C=O while increasing the polarity and hydrophilicity of the biochar surface. This modification of surface properties enhances the adsorption mechanism for heavy metals via ion exchange, electrostatic attraction, and surface complexation. However, for organic contaminants, the increased aromaticity and porosity of activated biochar improve π–π interactions and van der Waals forces [64].

### 5.2. Chemical Activation

Chemical activation is one of the most effective strategies to enhance the physicochemical properties of biochar and improving its adsorption performance [25]. This process involves impregnating the biomass or pre-formed biochar with activating agents such as acids (e.g., H_2_SO_4_, HCl, H_2_NO_3_, H_3_PO_4_), bases (e.g., KOH, NaOH), metal oxides (e.g., Fe_3_O_4_ and MgO), and salts (e.g., K_2_SO_4_, ZnCl_2_) [41,49]. Different methods are used to chemically activate biochar, including washing it with chemicals or directly incorporating chemicals into the biochar. Activation of biochar with acid helps to remove impurities and improve the acidic functional groups on its surface. For instance, the carboxylic, phenolic, and lactonic groups on the surface of biochar made from bamboo have been improved using nitric acid [21]. The surface area of biochar can be altered by acid modification, and the impact on surface area varies depending on the type and concentration of the acid used. An amount of 1 M of HCl acid-activated biochar derived from reeds increased the surface area from 58.75 to 88.35 m^2^/g [65]. The introduced chemicals promote dehydrogenation, dehydration, and partial oxidation during the thermochemical process, significantly enhancing microporous and mesoporous structures while substantially increasing the specific surface area [66]. The activation temperature ranges from 450 to 900 °C, which is lower than the temperature used for physical activation.

Leng et al. [25] stated that chemically activated biochar features well-developed micropores and a larger surface area than physically activated biochar. Conversely, chemically activated biochar usually has a relatively low yield and bulk density. In contrast, alkali modification with chemicals such as NaOH or KOH increases the porosity and surface area of biochar by increasing the number of negatively charged sites, thereby boosting the adsorption of positively charged metal ions [67]. Through chemical etching and gasification process, KOH activation increases microporosity, while H_3_PO_4_ activation facilitates crosslinking within the carbon matrix and introduces phosphate-containing groups that improve surface acidity and structural stability. For instance, Nguyen et al. [40] reported that bamboo-derived biochar at 600 °C had a specific surface area of 24.9 m^2^/g, but after activation with KOH, the surface area increased to 457 m^2^/g at a ratio of 1:8 and further increased to 913 m^2^/g at a ratio of 1.1. Another study by Zhao et al. [68] showed that activating biochar with ZnCl_2_ serves as a dehydrating agent, facilitating mesopore formation and increasing the adsorption capacity of organic pollutants. This kind of activation enhances the biochar surface by introducing functional groups such as COOH, -OH, and PO_4_, which improve electrostatic attraction, ion exchange, and surface complexation with heavy metals, while also facilitating π–π interactions and hydrogen bonding with organic pollutants.

**Figure 7:**
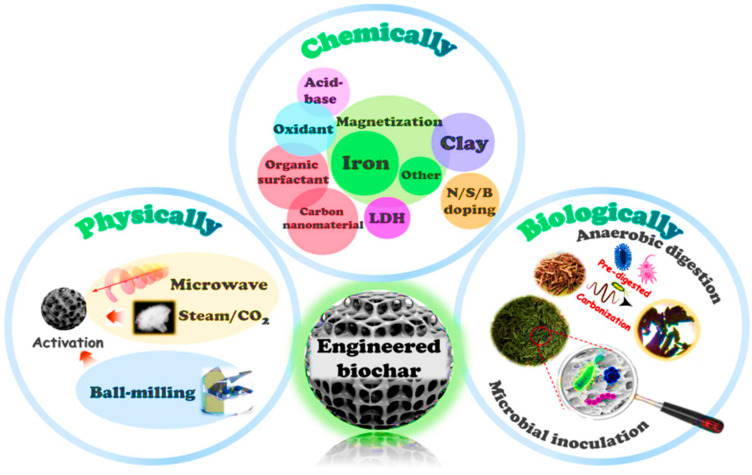
Biochar activation methods. Reproduced with permission from Chen et al. [73].

In addition to the above chemicals, functionalization of biochar with biochar-based composites has attracted increasing attention as a means to enhance its surface reactivity and adsorption capacity [69]. Various biochar-based composites have been developed to enhance biochar performance. For instance, metal-impregnated biochars, such as Fe, MgO, and ZnO-biochar, exhibit improved adsorption capacity due to the introduction of reactive metals and oxides that facilitate redox reactions, surface complexation, and ion exchange [69]. Furthermore, the addition of metal ions leads to the formation of nanoparticles and oxides on the biochar surface, acting as a physical barrier that protects the biochar from oxidation and enhances its stability [70]. Furthermore, magnetic biochar produced by incorporating iron oxides such as Fe_2_O_4_ or Fe_3_O_4_ into the carbon framework has attracted interest due to its significant catalytic redox potential, adsorption capacity, and magnetic separation properties. Magnetic particles have a dual purpose of providing active sites for electron transfer and metal complexation, as well as inhibiting particle agglomeration, which improves regeneration and reusability [71]. Murtaza et al. [72] reported that doping biochar with non-metal elements such as nitrogen (N), phosphorus (P), boron (B), sulfur (S), and others has become another efficient method to modify its surface chemistry and electronic structure, thereby enhancing adsorption and catalytic properties. Introducing such heteroatoms into the carbon structure during pyrolysis or post-treatment alters surface polarity and density, creating new binding sites. Figure 7 shows the biochar activation method.

## 6. Environmental Applications of Biochar in Wastewater Treatment

Biochar contains significantly more carbon content than commercially available activated carbon, making it a valuable tool in environmental management and pollution control [41]. Numerous studies have shown that biochar can effectively remove a various of pollutants from wastewater [16,18,29,37]. The following sections discusses the removal efficiency and adsorption performance of biochar for different environmental pollutants.

### 6.1. Application of Biochar in Removing Heavy Metals

Heavy metals are a group of metals with densities at least five times that of water, and they can be harmful or poisonous to both humans and the environment. This group includes elements such as cadmium (Cd), lead (Pb), arsenic (As), cobalt (Co), mercury (Hg), copper (Cu), zinc (Zn), nickel (Ni), iron (Fe), and others [30]. Among these, heavy metals like Cd, Cu, Zn, and Pb are toxic and carcinogenic, leading to various health issues, including allergies, skin irritation, headaches, tumors, and other diseases [11,64]. Heavy metal pollution is a major global environmental and public health challenge that urgently requires cost-effective, environmentally friendly, and sustainable removal technologies [18,24]. Because of their persistence and toxic nature, metals like Pb, Cd, Hg, and Cr pose hazardous effects on human health even at low concentrations [74].

The common methods for removing heavy metals include filtration, ion exchange, chemical precipitation, oxidation/reduction, and membrane separation [18,67]. However, these methods can be expensive, ineffective, and have some drawbacks. Thus, the researchers have identified an effective, eco-friendly and reasonably priced adsorbent material known as biochar for removing heavy metals from water. Biochar is highly effective at removing contaminants from aqueous solutions because of its large surface area, porous structure, and functional groups [11]. Biochar with a higher concentration of oxygen-containing functional groups can be produced at lower pyrolysis temperatures, and is particularly effective at removing inorganic contaminants [18].

**Figure 8:**
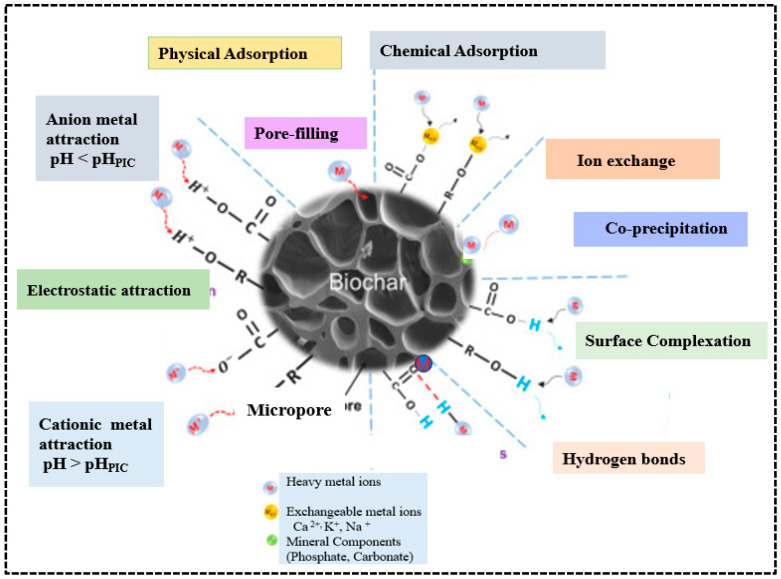
Mechanisms of adsorption of heavy metals on biochar. Reproduced with permission from Wang et al. [77].

Among various methods for removing heavy metals from water and wastewater, adsorption is considered the most effective. The main mechanisms for heavy metal removal include surface complexation between functional groups, ion exchange and precipitation [42]. Surface complexation happens when metal ions create coordination bonds with functional groups such as carboxyl (-COOH), hydroxyl (-OH), and carbonyl (-C=O). Ion exchange occurs when exchangeable cations on the biochar surface, such as Ca^2+^, K^+^, and Na^+^, are replaced with heavy metal ions from solution. Electrostatic attraction is another crucial mechanism of heavy metal adsorption occurs when the surface of biochar promotes metal ion adsorption, which varies with pH conditions. Additionally, physical adsorption via pore filling, hydrogen bonding, and co-precipitation with minerals such as phosphate and carbonates are further supporting mechanisms for heavy metal adsorption [18]. Together, all these mechanisms give biochar a strong affinity and high capacity for removing heavy metals from aqueous environments (Figure 8). Metallic ions can be physically adsorbed onto the biochar surface and trapped within its pores; therefore, biochar with a larger surface area and greater pore volume exhibits a higher affinity for heavy metals [75]. Due to their ligands and electrostatic forces, many biochar surfaces are negatively charged and can absorb positively charged metals [18]. Additionally, biochar interacts with heavy metal complexes or precipitates of their solid mineral phases and different functional groups. These properties make biochar an effective adsorbent and a promising low-cost alternative for removing heavy metals compared to activated carbon [76]. Adsorption is the accumulation of one or more fluids (liquids or gases) on the surface of a solid material, involving both chemical and physical processes. During adsorption, chemical species (molecules, atoms, or ions) interact at the interface of different phases.

Biochar’s physicochemical properties influence its porous structure, thereby improving its capacity to remove heavy metals [24]. In addition to this, biochar’s immobilization qualities can be beneficial for chemically modifying heavy metals, including surface functional groups, pH, and cation exchange capacity [18]. Characterization techniques have thus revealed that biochar exhibits significant adsorption efficiency for heavy metals [24]. The following Table 5 shows that different biomass-based adsorbents exhibit high removal efficiencies for several heavy metals.

#### Factors Affecting Removal Efficiency of Heavy Metals

Biochar removal efficiency of heavy metals is influenced by factors such as feedstock type, activation methods, surface properties, environmental conditions, and operational parameters [36]. For example, biochar produced from mineral-rich biomass, such as agricultural residues or animal manure, exhibits enhanced metal adsorption due to the presence of oxygen functional groups and alkaline minerals, which facilitate ion exchange and surface complexation. In contrast, biochar derived from lignocellulosic materials like wood depends on its surface area and porosity for effective physical adsorption [66]. Heavy metal characteristics like variations in ionic radius, valence, and affinity for surface functional groups lead to varying biochar capacity to remove different heavy metals. For instance, Liang et al. [80] reported that Pb is adsorbed more effectively than Cu and Zn due to stronger inner-sphere complexes with carboxyl groups on the biochar surface. Wang et al. [21] reported that activating biochar with chemicals such as HCl and KOH can significantly modify surface chemistry and pore structure, which improves adsorption capacity and selectivity for various contaminants. Biochar adsorption capacity increases with dosage up to a threshold level, beyond which excessive biochar can reduce effective surface area due to particle aggregation, which can reduce adsorption capacity [89]. For instance, the research conducted by Meng et al. [62], indicated a correlation between the initial removal rate of Cu^2+^ and the amount of pig manure in biochar. The removal efficiency of Cu^2+^ reached 95% at a biochar concentration of 5 g/L. While the adsorption capacity of biochar decreased from 12 to 6 mg/g, the removal rate of Cu^2+^ remained unchanged when the concentration of biochar was raised to 10 g/L. The researchers also revealed that the initial concentration of heavy metals affects the adsorption efficiency of biochar. The strength of heavy metal ion adsorption on biochar increases with the initial concentration of the ions. The cause of this effect is that heavy metal ions only absorb on the surface when their concentration is low [36]. Environmental conditions such as pH, temperature, and other competing ions affect the adsorption by affecting ionization, site availability, and electrostatic interactions [36]. Generally, factors that influence the adsorption capacity of biochar in wastewater treatment has been summarized in the following Table 6.

### 6.2. Application of Biochar in Removing Organic Pollutants

#### 6.2.1. Application of Biochar in Removing Persistent Organic Pollutants (POPs)

Organic pollutants encompass a wide variety of hazardous compounds, including organochlorine pesticides (e.g., DDT, aldrin, dieldrin, endrin, chlordane, and heptachlor), polycyclic aromatic hydrocarbons (PAHs) (e.g., anthracene, phenanthrene, naphthalene, p-nitrotoluene, pyrene) [90], volatile organic compounds (VOCs) (e.g., trichloroethylene, butanol, hexane, furan), polychlorinated biphenyls (PCBs) (e.g., PCB-1, PCB-18, PCB-28, PCB-52, PCB-101, PCB-118, PCB-138, PCB-153, PCB-180, PCB-209), synthetic dyes (e.g., methylene blue, Remazol Brilliant Blue, Basic Blue 18 and 9, Vat Red 10,Vat Orange 11, Crystal violet, Congo red, Acid blue 193, Acid yellow 36, Acid red) [91,92,93,94], and others. Additionally, several organic contaminants are specific to certain waste streams, such as estrogen compounds in sewage and animal manure, biomass degradation inhibitors like hydroxymethyl furfural (HMF), and phenolic and furan derivatives found in industrial effluents and landfill leachate [34,42]. These pollutants are of concern due to their persistence, toxicity, and bioaccumulation, which pose a significant risk to ecosystems and human health.

To establish a safe and sustainable water environment, effective treatment methods are necessary, as these pollutants pose serious risks to human health and the environment. Biochar has demonstrated strong potential for removing these pollutants through various mechanisms, including pore filling, hydrogen bonding, π–π interactions, hydrophobic interactions, ion exchange, and electron transfer [17]. The removal of organic contaminants mainly influenced by their interactions with the physicochemical properties of biochar [1]. Adsorption occurs through chemisorption (involving electrophilic interaction) and physisorption processes, such as pore diffusion, hydrophobic attraction, electrostatic forces, π–π electron donor–acceptor interactions, and hydrogen bonding. These mechanism are facilitated by functional groups such as carboxyl (-COOH), hydroxyl (-OH), and alkoxy (-ROH), which provide active sites for pollutant binding [34,42].

As noted earlier, the surface area and microporosity of biochar increase with pyrolysis temperature, making high-temperature biochar particularly effective for removing nonpolar organic contaminants. Conversely, biochar produced at lower temperatures lacks these characteristics. Biochar produced at lower temperatures (<500 °C) tends to have more oxygen and hydrogen-containing functional groups, which enhance affinity for polar organic molecules [34]. Several studies have examined the performance of various biochars in removing organic contaminants from wastewater [1,34]. For instance, Maletic et al. [95] and Odinga et al. [4] reported that biochar derived from pine wood and pine cones effectively adsorbed naphthalene, a common PAH. Similarly, Chai et al. [96] found that biochar made from woodchips and corn stover removed polychlorinated dibenzo-p-dioxins by 40% and 52.3%, respectively. Mandal et al. [97] observed that biochar prepared from agricultural wastes removed atrazine and imidacloprid pesticides with efficiencies ranging from 37.5–70.7% and 39.9–77.8%, respectively. Furthermore, perfluorooctane sulfonate was removed by 41% and 70% using biochar prepared from willow and maize straw respectively [98].

#### 6.2.2. Application of Biochar in Removing Antibiotics

Antibiotics are widely used to treat and prevent infections in both humans and animals. According to Li et al. [8], antibiotic use has increased over time, and it is predicted that global antibiotic consumption will rise by 67% by 2030 compared to 34.8 billion tons in 2015. Their persistence and bioaccumulation in the environment pose a significant threat to human health and the ecosystem [9,10,91]. Municipal wastewater is a primary source of antibiotics in surface and groundwater, with common compounds including sulfonamides, oxytetracycline, tetracyclines, quinolones, sulfamethazine, ciprofloxacin, clarithromycin, norfloxacin, macrolides, sulfamethoxazole, and enrofloxacin [8,92]. According to Dong et al. [9] and Zou et al. [10], antibiotic concentrations in wastewater range from several ng/L to several thousand ng/L. In particular, widely used antibiotics like tetracyclines, sulfonamides, and macrolides can have typical concentrations of up to 1000 ng/L [9].

The accumulation of antibiotics in aquatic ecosystems can promote the development of antibiotic-resistant genes, which combined with other pollutants, pose severe risk to both humans and aquatic life [8,9,10,91]. Thus, to mitigate these effects various wastewater treatment methods have been explored, including photolysis, adsorption, chemical oxidation, and biodegradation. Among these, adsorption using biochar is highly effective, affordable, and produce slow secondary pollution [8]. Several scholars have reported biochar’s effectiveness in removing antibiotics from wastewater. For instance, Jung et al. [99] reported that acetaminophen and naproxen were removed by 94.1% and 97.7% respectively, using biochar derived from pine chips, while Mondel et al. [100], found that steam activated derived biochar removes ibuprofen by 99.16%. In addition Yao et al. [101] also achieved 87.87% removal of Levofloxacin using biochar made from pomelo peel. In addition to the above examples, several studies have investigated the effectiveness of biochar derived from different feedstocks in removing various organic pollutants from wastewater. Table 7 summarizes the research findings on the applications of biochar in removing diverse classes of organic pollutants, including persistent organic pollutants, pesticides, pharmaceuticals, dyes, PAHs, and others.

#### 6.2.3. Application of Biochar in Removing Dye

Dyes are colored substances used to impart color to materials such as fabrics, paper, leather, plastics and even biological tissue [106,107]. They interact with the material surfaces through chemical or physical bonding since they are soluble in water and other solvents. Common examples include methylene blue, Remazol Brilliant Blue, Basic Blue 18 and 9, Vat Red 10, Vat Orange 11, Crystal violet, Congo red, Acid blue 193, Acid yellow 36, and Acid red [106]. Dyes, also known as colorants, are widely used across industries, such as textiles, plastics, food processing, cosmetics, rubber, printing, leather, and tanning [106]. However, wastewater from these industries contains toxic dyes that pose significant risks to the environment, aquatic life, and human health [108]. The discharge of dyes into the aquatic environment reduces light penetration, inhibiting photosynthetic activity in aquatic plants and algae [91]. Additionally, many dyes contain heavy metals and aromatic rings, making them persistent, bioaccumulate, and toxic to aquatic species. Some have been linked with carcinogenic, mutagenic, or teratogenic effects in various fish species and other organisms [107]. In humans, long-term exposure to dye-contaminated water can cause kidney, liver, brain, reproductive, and nervous system diseases [106]. Therefore, it is crucial to properly treat wastewater containing dyes before its release into water bodies [107].

According to Yagub et al. [106], approximately 7 × 10^5^ tons of dye-related materials and over 100,000 commercial dyes are produced annually. However, around 20% of synthetic dyes end up in industrial effluent, and 12% are lost during production and processing, contributing significantly to environmental contamination [107]. It is also estimated that the global textile industry utilizes more than 10,000 tons of dyes each year, with over 100 tons released into water streams. Several techniques are employed to remove dyes from wastewater, which include adsorption, advanced oxidation, coagulation, and membrane separation [98,99]. Among these, adsorption using biochar has emerged as one of the most effective technologies, owing to its high efficiency, rapid processing time, cost-effectiveness, and versatility [91,98,99]. Several studies have confirmed the efficiency of biochar in removing dyes from wastewater [83,98,99]. For instance, Elhamid et al. [109] reported that biochar obtained from rice straw achieved an adsorption capacity of 90.91 mg/g for methylene blue (MB) and 44.64 mg/g for crystal violet (CV) dyes. Similarly, tea leaf-activated biochar exhibited an adsorption capacity of 256.5 mg/g for malachite green, corresponding to 94% removal efficiency. Wu et al. [107] found that biochar produced from litchi peel exhibited exceptionally high adsorption capacities 404.4 mg/g for Congo red and 2468 mg/g for malachite green. In addition, Sartape et al. [92] reported that biochar made from wood apple shells had an adsorption capacity of 80.6 mg/g for malachite green. In addition to the above, Table 8 summarizes the effectiveness of biochar in removing different types of dyes, showcasing its potential as an efficient and sustainable adsorbent for dyes.

## 7. Conclusions and Future Perspective

This paper provides a comprehensive review of biochar preparation, modification, characterization techniques, and its applications in wastewater treatment. Traditional methods may not effectively remove pollutants, motivating the exploration of alternative materials such as biochar. Its high porosity, abundant surface functional groups, and ability to be engineered for better performance make biochar a promising material for environmental remediation. Biochar can be produced through various thermochemical methods, including pyrolysis, hydrothermal carbonization, gasification, and torrefaction, with each method influencing its structural and chemical characteristics. Higher pyrolysis temperatures, for example, enhance carbon content and porosity of biochar. Various factors, including feedstock type, pyrolysis temperature, activation method, and residence time can influence biochar physicochemical and adsorption capacity. Biochar modification and careful selection of feedstock and operating conditions further increase pollutant removal efficiency. Comprehensive characterization of biochar is essential to understand its structural, chemical, and functional attributes, which determine its potential applications in environmental remediation. Despite its potential, challenges remain regarding long-term stability, scalability, and environmental safety, due to the potential release of environmental pollutants associated with excessive use, which could lead to ecological problems. To ensure the sustainable use of biochar for wastewater treatment, future research should focus on enhancing biochar properties to improve pollutant removal, evaluating the lifecycle, developing regeneration and reuse strategies, and establishing quality standards for environmental applications.

## Figures and Tables

**Table 1:** Top 15 countries that publish on biochar applications.

S. No	Countries/Regions	Total Articles	Total Documents (%)
1	China	11,466	67.77
2	Usa	1922	11.36
3	India	1401	8.28
4	Pakistan	1005	5.94
5	Australia	940	5.56
6	South Korea	698	4.13
7	Germany	684	4.04
8	Saudi Arabia	657	3.88
9	Canada	613	3.62
10	Brazil	518	3.06
11	Italy	498	2.94
12	Egypt	480	2.84
13	Spain	475	2.81
14	Iran	459	2.71
15	Malaysia	416	2.46

**Table 2:** Different thermochemical conversion techniques with their typical yield.

S.N	Process	Time	Temp (°C)	Solid BC (%)	Liquid BC (%)	Gas BC (%)	Source
1	Pyrolysis						[40]
	Slow	Long	Low (300–700 °C)	30	35	35	
	Intermediate	Moderate	Intermediate (500 °C)	25	50	25	
	Fast	Short	High (500–1000 °C)	12	75	13	
2	Hydrothermal Carbonization	1–12 h	200–250 °C	50–80	5–20	2–5	
3	Gasification	Long residence time	High T °C > 700 °C	10	5	85	
4	Flash carbonization	<30 min, elevated	350–650 °C	50	NA	50	[37]
5	Torrefaction	Slow (<10 °C/min)	200–300 °C	80	0	20	[20]

Note: BC: biochar, NA: not available.

**Table 3:** Changes in composition of gas based on gasifying agent.

Gasification Agent	Syngas Composition (vol%–%)				Reference
	H_2_	CO_2_	CH_4_	N_2_	[45]
Oxygen	30–34	25–29	4–6	-	
Air	9–10	14–17	2–4	56–59	
Steam/CO_2_	24–50	10–19	5–12	-	

**Table 4:** Comparison of different torrefaction techniques.

Torrefaction Types	Time (Min)	Pressure (atm)	Temp (°C)	Pre-Drying	Moisture Handing	Solid Mass Yield
Dry torrefaction	10–240	1	200–300	Yes	Low	Higher
Wet torrefaction	5–240	2–200	180–260	No	High	Lower
Steam torrefaction	5–120	1–40	200–400	No	Higher	Lower
Microwave torrefaction	2.5–15	1	200–300	Yes	Low	Middle

**Table 5:** Applications of biochar in removing heavy metals and their removal efficiencies in literatures.

Biomass Used	Heavy Metal	Removal Efficiency (%)	Reference
Orang, banana peel, and rice husk	As	100	[29]
Rice husk	As	90.70	[78]
Potato peel and rice husk	As	>90%	[79]
Dairy manure	Pb	97.4	[80]
	Cu	53.3	
	Zn	54.5	
*Posiadonia oceanica* leaf sheaths	Pb	90	[81]
Wheat	Cd	99	[82]
Oilseed rape	Cd	98.49	
*Miscanthus*	Cd	99	
Dairy manure	Cd	96.86	[53]
	Zn	56.48	
	Cu	80.58	
Pineapple peel	Cr(VI)	100	[83]
Waste walnut shells	Ni	80	[84]
Broiler litter	Cu^2+^	75	[85]
Broiler litter	Cd^2+^	22	[85]
*Ficus microcarpa*	U(VI)	82	[86]
Pine needle	Hg	85	[48]
Coconut shell	Pb^2+^	92	[87]
Dairy manure	Pb^2+^	89	[87]
Hardwood	Cu^2+^	58	[87]
Activated corn stover	Zn^2+^	95	[85]
	Ni^2+^	96	
Soybean straw	Zn^2+^	48	[85]
Sesame straw	Cr^2+^	67	[88]
Activated broiler litter	Zn^2+^	39	[85]
Soybean straw	Cd^2+^	54	[85]

**Table 6:** Factors influencing adsorption capacity of biochar.

Factors	Effect	Mechanism	Reference
Temperature	Enhance adsorption up to the optimal point	Higher temperature enhances diffusion and promotes endothermic adsorption; excessive heat may degrade active sites	[63]
pH	Significantly affects the adsorption capacity	Change surface ionization state and surface charge of biochar, low pH favors cation adsorption, while high pH favors anion adsorption	[54]
Feedstock type	Affects adsorption capacity and mechanism	Mineral content, surface area, and functional groups of feedstocks determine	[66]
Initial pollutant concentration	Higher concentration increases adsorption capacity but reduces overall removal percentage	Creates stronger concentration gradient; may reach equilibrium faster at lower efficiency	[36]
Biochar dosage	Increases removal efficiency until reaching a saturation level	Provides more adsorption sites; above optimum, aggregation reduces available surface area and adsorption capacity reduced	[89]
Contact Time	Rapid adsorption initially, followed by equilibrium	Fast surface binding occurs first; later stages governed by intraparticle diffusion	[36]
Ionic Strength	Variable effect depending on pollutant charge	Competing ions interfere with electrostatic adsorption; high ionic strength often decreases removal of charged species	[80]
Surface Functional Groups	Correlates with adsorption efficiency	-OH, -COOH, and -C=O groups promote complexation, ion exchange, and hydrogen bonding	[21]

**Table 7:** Various research results on application of biochar in removing different organic pollutants.

Feedstock Source	Target Compound	Preparation Methods	Removal Efficiency (%)	Reference
Rice	Pentachlorophenol	Pyrolysis	96	[3]
Hardwood	Poly aromatic hydrocarbons	Pyrolysis	32	[5]
Mung bean husk	Ibuprofen	Pyrolysis	100	[100]
Pistachio shell	Phenol	Pyrolysis	51	[102]
Peanut shell	Trichloroethylene	Pyrolysis	100	[103]
Wood chips	Polychlorinated dibenzo-p-dioxins	Pyrolysis	40	[96]
Corn Stover	Polychlorinated dibenzo-p-dioxins	Pyrolysis	52.3	[96]
Maize straw	Perfluoro octane sulfonate	Pyrolysis	70	[98]
Willow	Perfluoro octane sulfonate	Pyrolysis	41	[98]
Pine chips	Acetaminophen	Pyrolysis	94.1	[99]
Pine chips	Naproxen	Pyrolysis	97.7	[99]
Biogas residue	Norffoxacin	Pyrolysis	91.47	[104]
Pomelo peel	Levoffoxacin	Pyrolysis	87.87	[101]
Soybean stalk	Phenanthrene	Pyrolysis	99.5	[105]
Reeds	Pentachlorophenol	Pyrolysis	43–100	[8]

**Table 8:** Different research results on application of biochar for removal of dyes.

Dyes	Biomass Used	Removal Efficiency (%)	Reference
Methylene blue	Tobacco Stem Ash	60–81	[94]
Congo red	Pine cone	60.5–75.5	[110]
Methylene blue	Pine cone	63.83–94.82	[111]
Apricot seed	Astrazone Black	91–62	[112]
Sugarcane bagasse	Rhodamin e B	87.1–99.1	[113]
Sugarcane bagasse	Basic blue 9	55.5–94	[113]
Rice straw	Crystal Violet	92.7	[109]
Modified Mango seed	Methylene Blue	96–99.9	[109]
Rice straw	Methylene Blue	94.45	[109]

## Data Availability

No new data were created in this review.

## Abbreviations

The following abbreviations are used in this manuscript:

BC	Biochar
BET	Brunauer–Emmett–Teller analysis
FTIR	Fourier transform infrared spectroscopy
OCPs	Organochlorine pesticides
POPs	Persistent organic pollutants
PAHs	Polyaromatic hydrocarbons
PCBs	Polychlorinated biphenyls
SEM	Scanning electron microscopy
TGA	Thermogravimetric analysis
XPS	X-ray photoelectron spectrometry
XRD	X-ray diffraction
